# Similarities and Differences Between Staphylococcal and Streptococcal Toxic Shock Syndromes in Children: Results From a 30-Case Cohort

**DOI:** 10.3389/fped.2018.00360

**Published:** 2018-11-28

**Authors:** Etienne Javouhey, Pierre-Adrien Bolze, Claire Jamen, Gerard Lina, Cédric Badiou, Claire Poyart, Aurelie Portefaix, Anne Tristan, Frédéric Laurent, Michèle Bes, François Vandenesch, Yves Gilletand, Olivier Dauwalder

**Affiliations:** ^1^Pediatric Emergency and Critical Care Unit, Hospices Civils de Lyon, Hôpital Femme Mère Enfant, Bron, France; ^2^Faculté de Médecine Lyon Est, Université de Lyon, Domaine de la Buire, Lyon, France; ^3^Service de Gynécologie et Obstétrique, Hospices Civils de Lyon, Hôpital Femme Mère Enfant, Bron, France; ^4^Centre National de Référence des Staphylocoques, Institut des Agents Infectieux, Centre de Biologie et de Pathologie Nord, Hospices Civils de Lyon, Lyon, France; ^5^Laboratoire de Bactériologie, Centre de Biologie et de Pathologie Nord, Institut des Agents Infectieux, Hospices Civils de Lyon, Lyon, France; ^6^Centre International de Recherche en Infectiologie (CIRI), Inserm U1111, Université Lyon 1, Ecole Normale Supérieure de Lyon, CNRS UMR5308, Lyon, France; ^7^Centre National de Références des Streptocoques - Groupe Hospitalier Paris Centre Cochin-Hôtel Dieu-Broca, Assistance Publique Hôpitaux de Paris, Paris, France; ^8^Institut Cochin Université Sorbonne Paris Descartes, Paris, France; ^9^INSERM 1016, Institut Cochin, Paris, France; ^10^CNRS, UMR8104, Institut Cochin, Paris, France; ^11^EPICIME-CIC 1407 de Lyon, Inserm, Service de Pharmacotoxicologie, Hospices Civils de Lyon, Bron, France

**Keywords:** toxic shock syndrome, children, *Staphylococcus aureus*, *Streptococcus pyogenes*, Vβ T-cell signature, antitoxin therapy

## Abstract

**Introduction:** Toxic shock syndromes (TSS) are severe shocks due to staphylococcal or streptococcal infection that require specific treatments. The early recognition of these shocks is crucial to improve their outcomes.

**Objectives:** The primary objective of this study was to compare characteristics and outcomes of staphylococcal and streptococcal TSS in children, in order to identify putative early clinical diagnostic criteria. Secondary objectives were to determine the toxin gene profiles of associated isolated strains and the relevance of measuring Vβ T-cell signatures to confirm the diagnosis.

**Study design:** We performed a multicenter retrospective evaluation of clinical data, biological results, and treatment outcomes of children with a confirmed or probable case of staphylococcal or streptococcal TSS. Children were consecutively included if they were admitted to the pediatric intensive care units of Lyon (France), between January 2005 and July 2011.

**Results:** Among the 30 analyzed children, 15 presented staphylococcal TSS and 15 streptococcal TSS. The most frequent origin of staphylococcal and streptococcal TSS was the lower respiratory tract (53%) and the genital tract (47%) respectively. Non-menstrual TSS syndrome cases presented more frequently with neurological alterations, and digestive signs were predominant in menstrual forms. Compared to Staphylococcal TSS, Streptococcal TSS presented with higher organ dysfunction scores (median Pediatric Index of Mortality 2 score 20.9 (4.1–100) vs. 1.7 (1.3–2.3), *p* = 0.001), required respiratory support more frequently (80 vs. 33%, *p* = 0.02), were intubated for a longer time (3 days (0.75–5) vs. 1 day (0–1.5), *p* = 0.006) and had a non-significant trend of higher, case-fatality rate (20 vs. 7%, *p* = 0.60). The lack of antitoxin therapy was associated with higher case-fatality rate (50 vs. 4%, *p* = 0.04). The Vβ repertoire measurements exhibited toxin dependent-alterations in accordance with the toxin gene profiles of isolated strains in both types of toxic shock syndromes. Regarding toxin gene profiles of isolated strains, 10/15 *Staphylococcus aureus* belonged to clonal complex (CC) 30 and 6/12 *Streptococcus pyogenes* were *emm1* type suggesting clonal etiologies for both staphylococcal and streptococcal TSS.

**Conclusion:** Despite the involvement of functionally similar toxins, staphylococcal and streptococcal TSS differed by their clinical signs, origin of infection and prognosis. The detection of Vβ profiles was useful to confirm the diagnosis of staphylococcal and streptococcal TSS and for the identification of involved toxins.

## Introduction

Toxic Shock Syndrome (TSS) is a severe acute illness characterized by high fever, hypotension, rash, multi-organ system dysfunction, and desquamation during convalescence. TSS is caused by toxin-producing strains of *Staphylococcus aureus* or *Streptococcus pyogenes* and occurs in both adult and pediatric patients (1–5). TSS remains a rare but severe disease, with a mortality rate that varies from 4 to 27% for streptococcal (Str) TSS (2–4) and from 0 to 22% for menstrual and non-menstrual staphylococcal (Sta) TSS (1). Studies conducted in pediatric intensive care units (PICU) reported a mortality rate that can reach 25% for Str-TSS (2–6). The outcome of Sta-TSS is more favorable in children than in adults (7, 8). TSSs have a specific pathophysiology linked to superantigen exotoxins. Superantigens (SAg), in contrast with conventional antigens, do not need to be processed by antigen-presenting cells before being presented to T cells. They instead directly stimulate T cells by cross-linking major histocompatibility complex class II molecules on the antigen-presenting cells with the variable portion of the T-cell antigen receptor chain (Vβ TCR), thereby inducing massive polyclonal cell proliferation (9, 10). Due to their structural differences, each SAg links preferentially to one or several Vβ repertoire, thus inducing targeted T cell expansion and the massive pro-inflammatory response (11). A transient depletion of targeted Vβ TCRs may be observed at the early phase of TSS due to the concomitant lymphopenia, to the mobilization/accumulation of T cells from peripheral blood to lymph nodes or to the spleen, and/or from downregulation of TCR molecules binding the toxin targeted Vβ repertoire(s) soon after T cell activation (12).

The diagnosis of TSS is based on the association of standardized clinical signs defined by the Centers for Disease Control (CDC), but some of them may be transient (i.e., hypotension), lacking (i.e., cutaneous rash) or of delayed occurrence (i.e., desquamation), and diagnosis is often difficult during the early stages of the diseases (13, 14). Distinction from septic shock, Kawasaki disease with shock, drug reaction with eosinophilia and systemic symptoms (DRESS) syndrome is sometimes difficult (15). Nevertheless, treatments of these differential diagnoses differ significantly and early recognition of these diseases is a key point for the prognosis. Because SAgs are active at very low concentrations (< 1 pg/mL) that are barely detectable *in vivo*, the identification of *ex vivo* specific Vβ TCR alterations could help to diagnose or to confirm TSS (16–26).

Early diagnosis of TSS is required because specific TSS treatments with antitoxin effects must be added (1, 27) to adapted antimicrobial chemotherapy: clindamycin, rifampicin or linezolid to reduce exotoxin synthesis (2, 9, 28, 29), and intravenous immunoglobulin (IVIG) therapy to neutralize the SAg, especially in severe TSS (1–4, 7, 8, 30–36). Because of severity of the disease, the clinical characteristics of Sta- and Str-TSS have been primarily described in adult patients.

The objectives of this pediatric study were to compare the characteristics and the outcome of staphylococcal and streptococcal TSS in children to identify putative early clinical diagnostic criteria; to identify factors associated with case-fatality; to study the toxin gene profiles of associated isolated strains; and to assess the relevance of measuring Vβ T-cell signatures to confirm the diagnosis.

## Materials and methods

### Patient selection

#### Selection of eligible TSS cases

The Sta- and Str-TSS cases were retrospectively selected by searching for the keywords “toxic” or “toxin-associated shock syndrome” in the hospital information system, as well as in the medical records and databases of the two PICUs of the Hospices Civils de Lyon, France (Debrousse Hospital and Edouard Herriot Hospital from 2005 to 2008), then the PICU of the “Hôpital Femme Mère Enfant” from 2008 to 2011 grouping those two units when they closed. A supplemental research was performed from the database of the French Staphylococcal and Streptococcal National Reference Centers (NRC) which collects prospectively strains involved in staphylococcal and streptococcal diseases, and notably toxin associated diseases. Appointed by Santé Publique France, an agency of the French Ministry of health, NRC missions include (i) strain expertises (identification, resistance, virulence, and typing), (ii) epidemiological surveillance of infections (i.e., increase of incidence, new virulent factor, etc.), and (iii) advice to public authorities, health agencies, and health professionals.

### Included cases

All records of the cases identified were reviewed by two authors (CJ, EJ) to check if they met the CDC criteria for confirmed and probable TSS cases (13, 14). Sta-TSS patients with all CDC criteria but desquamation were included in the probable Sta-TSS group, as this sign mainly occurred lately in the course of the disease and was therefore lacking in the PICU's medical record.

### Collected data

Clinical and biological data, including pediatric index of mortality (PIM 2), pediatric logistic organ dysfunction (PELOD) scores and 30 days outcomes (length of PICU stay and mortality) were collected anonymously (37, 38).

### Ethics statements

According to the French policies, our study was defined as a non-interventional study as it met the following two criteria: (i) parents of children included in the database have received information about the study and (ii) an non-formalized right of opposition was collected in the clinical records. Regarding this lack of written informed consent, patient records were collected anonymously and de-identified prior to analysis. Thus, this study was approved by an institutional ethics review board (“Comité de Protection des personnes Sud Est IV, DC-2008-176”).

### Immunological data

Blood samples were collected in EDTA tubes from 1 to 4 days (D) after PICU admission to measure Vβ CD3+T cell alterations. Due to the initial lymphopenia, the mobilization/accumulation of T cells from peripheral blood to lymph nodes or to the spleen, and/or from downregulation of TCR molecules binding the toxin targeted Vβ repertoire(s) soon after T cell activation, a second measurement was performed between D+3 and D+8 of PICU admission in case of massive decrease of one or association of Vβ repertoires known to be targetet by SAg. These measurements could only be performed 5 days a week (Monday–Friday) which explains that the first determination could range from D+1 to D+4 and the second from D+3 to D+8. The physicians responsible for case coding as well as those responsible for the clinical PICU's databases were not aware of the results of these immunological investigations.

The peripheral blood mononuclear cells were stained with CD3 and 24 TCR Vβ element antibodies (IO Test Beta Mark® kit, Beckman Coulter, Marseille, France) and analyzed with FACScan® flow cytometer (BD, Pont de Claix, France), as previously described (25). Based on our previous data that showed an association between TCR Vβ expansions and involved staphylococcal toxin, we limited the measure of Vβ CD3+T cell alterations to these 24 out of 65 known human Vβ elements.

### Microbiological data

Different anatomical sites were sampled and sent for microbiological analysis (culture and isolation of *S. aureus* or *S. pyogenes*) in order to identify etiology and source of infection. Strains were identified by biochemical tests, such as clumping factor, the coagulase test or Lancefield group agglutination (bioMérieux, Marcy l'Etoile, France), and with Phoenix® strips (BD, Pont de Claix, France) or mass spectrometry (VITEK MS®, BioMérieux, France). Antibiotic susceptibilities were determined using a Phoenix 100® instrument (BD) or by the disc diffusion method (SIRSCAN® I2A, Peyrols, France) following CA-SFM guidelines.

#### Toxin gene profiles of *S. aureus* strains determined by the French NRC of staphylococci

The toxin gene profiles of *S. aureus* isolates were characterized for all patients. Staphylococcal DNA was extracted (Qiagen, Courtaboeuf, France), and the toxin gene profile was determined using Identibac *S. aureus* Genotyping® DNA microarrays (Alere, Jouy en Josas, France), as previously described (39). This Identibac *S. aureus* Genotyping® DNA microarray screened genes for strains identification, virulence factors, resistance and allow for the assignment to a probable clonal complex (CC) (39).

#### Toxin gene profiles and M protein typing of *S. pyogenes* strains determined by the French NRC of streptococci

The toxin gene profiles of *S. pyogenes* isolates were characterized for 12/15 patients (three strains were not transmitted to the French NRC for streptococci). As previously described, *S. pyogenes* strains were analyzed to determine their gene profiles of superantigenic toxins and M protein types (*emm*) (8) by PCR.

### Statistical analysis

Data were analyzed with SPSS for Windows version 17.0 (SPSS Inc., Chicago, Illinois, USA). Qualitative variables were compared with a Chi-Square test supplemented by Fisher's exact test for low effective groups (*n* < 5). Quantitative variables are reported as the means and standard deviation or as medians and quartiles 1 and 3 for the variables that had distributions that were not normal. The Kolmogorov-Smirnov test was used to study the normality of the distribution of the continuous variables. The quantitative variables were compared with Student's *t*-test or with a non-parametric Wilcoxon test depending on the normality of their distribution. *P*-values below 0.05 were considered to indicate statistical significance.

## Results

### Characteristics of the study population

Among the 34 patients identified from the cross-analysis of the databases, 30 (11 boys and 19 girls) were finally included in the study. Among them, 15 Str-TSS cases (11 confirmed and 4 probable cases according to the CDC criteria) and 15 Sta-TSS cases, 6 of which were menstrual Sta-TSS and 9 non-menstrual Sta-TSS (4 confirmed cases and 8 probable cases according to the CDC criteria) were included. The four excluded patients were 1 Stevens-Johnson syndrome, 1 Kawasaki syndrome with shock, 1 severe Panton-Valentine staphylococcal infection and 1 patient hospitalized in an intermediate care unit. The median age was 5.2 years (interquartile range: 1.4–12.8, Table 1). *S. pyogenes* were isolated in all the patients.

**Table 1:** Clinical and biological characteristics of patients with staphylococcal or streptococcal toxic shock syndrome.

		**Missing data**	**Sta-TSS^a^ (*n* = 15)**	**Str-TSS^b^ (*n* = 15)**	***p***
Demographical characteristics	Male (%^c^)	0	4 (27%)	7 (47%)	0.45^d^
	Age (years) median (Q1-Q3^e^)	0	12.8 (5–15.7)	1.7 (0.7–5.4)	0.001^f^,^*^
Hemodynamic characteristics	Hypotension (%)	0	15 (100%)	15 (100%)	1^g^
	Need of amine support (%)	0	10 (67%)	12 (80%)	0.68^g^
	Duration of treatment with amines (days) median (Q1-Q3)	0	1 (0–1.8)	2 (0.8–3)	0.17^f^
Pulmonary signs	${\text{FiO}}_{2}^{\text{h}}$ > 50% (%)	1	5/14 (36%)	13/15 (87%)	0.01^g^,^*^
	Requirement of mechanical ventilation (%)	0	5 (33%)	12 (80%)	0.02^g^,^*^
	Duration of intubation median (days) (Q1-Q3)	0	1 (0–1.5)	3 (0.75–5)	0.006^f^,^*^
	ARDS^i^ (%)	2	1/14 (7%)	5/14 (36%)	0.16^d^
Organ dysfunctions	Creatinine, maximum value (μmol/L) mean (SD^j^)	0	122.1 (115.8)	68.3 (49.6)	0.11^k^
	Liver alterations^l^ (%)	2	10/14 (71%)	8/14 (57%)	0.69^g^
	Number of organ dysfunction^l^ median (Q1-Q3)	0	3 (2–4)	4 (3–5)	0.13^f^
Cutaneous signs	Rash (%)	0	15 (100%)	11 (73%)	0.10^g^
	Desquamation (%)	6	5/13 (39%)	3/11 (27%)	0.68^d^
	Digestive signs (%)	0	11 (73%)	7 (47%)	0.26^g^
Inflammatory parameters	Fever ≥ 38.9°C (%)	0	15 (100%)	15 (100%)	1^g^
	Leukocytes, minimum value (G/L)mean (*SD*)	0	8.6 (3.7)	8.5 (6.3)	0.95^k^
	Leukocytes, maximum value (G/L) mean (*SD*)	0	14.6 (6.2)	22.3 (13)	0.049^k^,^*^
	Lymphocytes, minimum value (G/L) median (Q1-Q3)	2	0.2 (0.1–1.2)	0.6 (0.5–0.9)	0.056^f^
	C-Reactive Protein (mg/L) mean (SD)	2	164 (82.8)	238.8 (103.8)	0.044^k^,^*^
Hemostasis parameters	Platelets, minimum value (G/L) mean (SD)	0	124.8 (52.5)	140.2 (100.6)	0.61^k^
	Disseminated Intravascular Coagulation	1	8/15 (53%)	8/14 (57%)	1^g^
Severity scores and outcome	PIM2^m^ score median (Q1-Q3)	3	1.7 (1.3-2.3)	20.9 (4.1-100)	0.001^f^,^*^
	PELOD^n^ J1 score median (Q1-Q3)	1	11 (11-21)	16.5 (11.8–25)	0.15^f^
	Length of stay in ICU^°^ (days) median (Q1-Q3)	0	3 (2-4)	6 (3–9)	0.02^f^,^*^
	30 day death	0	1 (7%)	3 (20%)	0.60 ^d^

a *Sta-TSS, Staphylococcal Toxic Shock Syndrome*.

b *Str-TSS, Streptococcal Toxic Shock Syndrome*.

c *% percent of total population*.

d *Statistical analysis performed with Fisher exact test*.

e *Q1-Q3, Interquartile range*.

f *Statistical analysis performed with Wilcoxon test*.

g *Statistical analysis performed with Chi-square test*.

h *FiO_2_, Fraction of inspired Oxygen*.

i *ARDS, Acute Respiratory Distress Syndrome*.

j *SD, standard deviation*.

k *Statistical analysis performed with Student t-test*.

l *Liver alterations and organ dysfunctions were defined in CDC criteria for case definition of toxic shock syndrome (13, 14)*.

m *PIM2 Pediatric Index of Mortality 2 score*.

n *PELOD Pediatric Logistic Organs Dysfunctions score*.

° *ICU, Intensive Care Unit*.

* *Statistically significant data with the corresponding statistical test*.

### Comparison between patients with staphylococcal and streptococcal toxic shock syndrome

Children with Str-TSS were significantly younger than children with Sta-TSS [1.7 years (0.7-5.4) vs. 12.8 years (5–15.7), *p* = 0.001]. At day 1, compared to Sta-TSS patients, Str-TSS patients needed ventilation support more often and had longer ventilation duration [3 days (0.75–5) vs. 1 day (0–1.5), *p* = 0.006]; had higher leukocyte counts (22.3 vs. 14.6 G/L, *p* = 0.049) and significantly higher CRP levels (238.8+/– 103.8 vs. 164+/– 82.8 mg/l, *p* = 0.04, respectively) and presented with significantly higher PIM 2 score (median 20.9 (4.1–100) vs. 1.7 (1.3–2.3), *p* = 0.001) Other clinical variable were similar in the two groups.

The primary origin of the Str-TSS was the lower respiratory tract (53% of Str-TSS cases) whereas menstrual and non-menstrual Sta-TSS involved the vagina, and the skin or the upper respiratory tract respectively (Table 2).

**Table 2:** Infectious source of patients with staphylococcal and streptococcal toxic shock syndrome.

**Etiological infectious sites**	**Staphylococcal**	**Streptococcal**	
	**TSS^a^**	**TSS**	***p*****^b^**
	**(*****n*** = **15)**	**(*****n*** = **15)**	
Bacteremia	1 (7%)	6 (40%)	0.08
Pleura or lung	1 (7%)	8 (53%)	0.01^*^
Postoperative TSS	0 (0%)	2 (13%)	0.48
Skin and soft tissues	4 (27%)	3 (20%)	1
Upper respiratory tract	3 (20%)	3 (20%)	1
Vagina	7 (47%)	0 (0%)	0.006^*^
Varicella superinfection	2 (13%)	3 (20%)	1
Indefinite	0 (0%)	1 (7%)	1

a *TSS, Toxic Shock Syndrome*;

b *Statistical analysis performed with Chi-square test*.

* *Statistically significant data (p < 0.05) with the corresponding statistical test*.

There was no difference between groups regarding IVIG use (53% for Str-TSS and 47% for Sta-TSS, *p* = 1) or the use of anti-infectious agents with antitoxin effects (clindamycin, linezolid, rifampicin; 93% for Str-TSS and 87% for Sta-TSS, *p* = 1).

### Comparison between menstrual and non-menstrual staphylococcal toxic shock syndrome

The most striking difference between menstrual and non-menstrual Sta-TSS was the constant presence of gastrointestinal signs (i.e., abdominal pain and emesis) in menstrual Sta-TSS (100 vs. 50%, *p* = 0.08) There was also a non-significant trend toward a higher frequency of neurological symptoms (i.e., confusion, impaired consciousness) in non-menstrual Sta-TSS (50 vs. 14%, *p* = 0.28).

### Factors associated with case-fatality:

Our retrospective study reported 4/30 deaths (13%) (Table 3). The non-survivors significantly presented with more organ dysfunctions, with a higher occurrence of acute respiratory distress syndrome and with a higher PELOD score measured at D+1. The case-fatality rate was not significantly different between the Staphylococcal and Streptococcal TSS groups (7 vs. 20% *p* = 0.60). All the children received adapted antimicrobial therapies for *S. aureus* or *S. pyogenes*, and 27/30 received an antitoxin therapy (clindamycin or clindamycin + IVIG) with a significant association with survival (Table 3). Among the 4 non-survivors, three died rapidly (one during the transport to PICU, one 4 h after arrival, one had presented a cardiac arrest just prior to PICU admission), and only one of them received an antitoxin antibiotic.

**Table 3:** Comparison of clinical characteristics and effects of antitoxin therapies on live vs. dead TSS cases.

	**Dead (*n* = 4)**	**Survivor (*n* = 26)**	***P***
Etiology of toxic shock syndrome			*p* = 0.5977
- Sta-TSS^a^ (%)^b^	1 (7%)	14 (93%)	
- Str-TSS^c^ (%)	3 (20%)	12 (80%)	
Number of organ dysfunctions (SD^d^)	5 (0, 8)	4 (1, 3)	*p =* 0.04^*^
ARDS^e^ (%)	4/4 (100%)	4/26 (15%)	*p =* 0.04^*^
PELOD^f^ score at D+1 (SD)	28 (13, 7)	12 (7, 1)	*p =* 0.04^*^
Antitoxin therapies (overall)			*p =* 0.04^*^
- No treatment (%)	2 (67%)	1 (33%)	
- At least one antitoxin therapies (clindamycin; clindamycin + IVIG^g^) (%)	2 (7%)	25 (93%)	
Antitoxin therapy: clindamycin only			*p =* 0.0813
- No treatment (%)	2 (67%)	1 (33%)	
- Clindamycin only (%)	1 (8%)	11 (92%)	
Antitoxin therapy: clindamycin + IVIG			*p =* 0.5977
- No treatment (%)	3 (20%)	12 (80%)	
- Clindamycin + IVIG (%)	1 (7%)	14 (93%)	
Antitoxin therapy: clindamycin + IVIG			*p =* 1.0000
- Clindamycin only (%)	1 (8%)	11 (92%)	
- Clindamycin + IVIG (%)	1 (7%)	14 (93%)	

a *Sta-TSS, Staphylococcal Toxic Shock Syndrome*.

b *%, percent of total population*.

c *Str-TSS, Streptococcal Toxic Shock Syndrome*.

d *SD, standard deviation*.

e *ARDS, Acute Respiratory Distress Syndrome*.

f *PELOD, Pediatric Logistic Organs Dysfunctions score*.

g *IVIG, Intravenous Immunoglobulin*.

* *Statistically significant data with the corresponding statistical test*.

### Microbiological features

*S. aureus* strains belonged to the accessory gene regulator (Agr)1 (2/15), Agr2 (2/15) or Agr3 (11/15) genetic backgrounds. According to DNA microarrays assignments, 10/15 belongs to clonal complex (CC) 30, 1/15 to CC5, 1/ 15 to CC45, 1/15 to CC22, and the sole MRSA strain is CC5 and belongs to Geraldine clone. All menstrual Sta-TSS strains contained the TSS toxin gene (*tst*) that encodes TSS toxin −1 (TSST-1), the staphylococcal enterotoxin A gene (*sea*) gene that encodes staphylococcal enterotoxin (SE) A (4/7), the enterotoxin gene clusters (*egc*) that encode SEG, SEI, staphylococcal enterotoxin like (SE*l*) M, SE*l*N and SE*l*O (5/7). The non-menstrual Sta-TSS isolates were characterized by the presence of the *tst* gene encoding TSST-1 (6/8), *seb* (1/8), or *sec* (1/8) in association with other toxin genes; the most frequently found toxin genes were *sea* (4/8) and *egc* (7/8). Only one strain involved in non-menstrual Sta-TSS was resistant to methicillin.

*S. pyogenes* strains were primarily types *emm*1 (6/12) or *emm*12 (2/12). Toxin gene analysis showed the following 4 different profiles: contained the streptococcal pyrogenic exotoxin A (*spe*A) and *spe*C genes encoding streptococcal pyrogenic exotoxin (SPE) A and SPEC, respectively (2/12), contained only the *spe*A gene (4/12), contained only *spe*C (5/12) or contained neither *spe*A nor *spe*C but did encode *spe*B (1/12). The gene *spe*B was present in all *S. pyogenes* strains included in our study.

### Immunological features: superantigenic toxins and Vβ T-cell signatures

**Figure 1:**
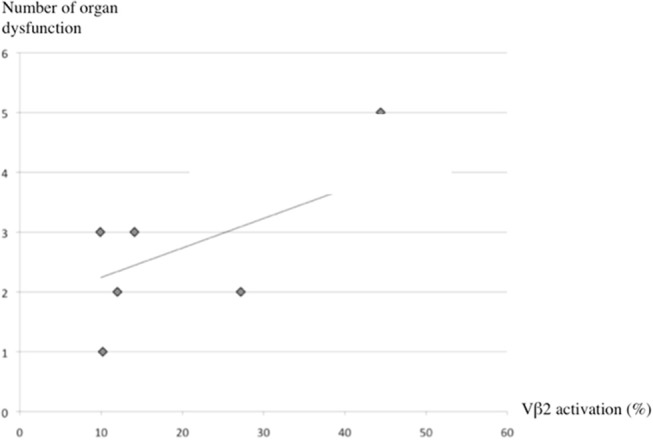
Correlation between organ dysfunctions and Vβ2 expression on CD3^+^ T cells. Correlation between the number of organ dysfunctions and the Vβ2 expression measured on CD3^+^ T cells between days 3 and 5 after the onset of staphylococcal toxic shock syndrome. Pearson's correlation (R^2^) was used to measure co-linearity between the selected independent variables.

The Vβ repertoire profile of CD3+ T cells (Vβ profile) was determined at D1-4 after PICU admission in a subset of 18/30 patients that included 12 Sta-TSS and 6 Str-TSS cases (Tables 4, 5). Targeted by TSST-1, a significant Vβ2 alteration was observed in all confirmed and probable TSS-Sta cases. Whereas, a Vβ2 increase was measured for 8/12 (67%) Sta-TSS patients, it was delayed for 4 Sta-TSS cases with initially a large decrease of Vβ2 repertoire followed by a large expansion at the second measurement performed between D3 and D5. A correlation between the number of organ dysfunctions and the level of Vβ2 expression on CD3+ T cells between days 3 and 5 post-Sta-TSS onset was found (Figure 1). Regarding 6 Str-TSS cases, expansions of some or all Vβ repertoires targeted by SPEA or SPEC were also measured in all confirmed and probable Str-TSS cases at the first determination (Table 5).

**Table 4:** Vβ T cell signatures and *Staphylococcus aureus* toxin gene profiles of patients with staphylococcal toxic shock syndrome.

		**Treatments**		**Immunological data**			**Microbiological data**				
	**Gender**	**Antitoxin antibiotic**	**IVIG^a^ (Dose)**	**Vβ alterations of CD3^+^ T cells (%)^b^**	**Vβ alterations of CD3^+^ T cells (%)^b^**	**Toxin suspected according to Vβ modification profile**	**Site of isolation – Infection or carriage strain**	**Methicillin suscepti-bility**	**Allele of Agr system**	**Toxin gene profile**	**Clonal complexe**
				**Measurement 1 [day post shock onset]**	**Measurement 2 [day post shock onset]**						
Menstrual staphylococcal toxic shocks	F^c^	Yes	Yes (NA^d^)	Vβ2 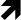 (44.4%) [D^e^+4]	ND^f^	TSST-1^g^	Vagina (c)^h^	MSSA^i^	3	*tst*^j^; *sea*^k^; *egc*^l^	CC30
	F	Yes	No	Vβ2 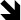 (1.1%) [D+1]	Vβ2 ↔ (10.2%) [D+5]	TSST-1	Vagina (c)	MSSA	1	*tst*; *egc*	CC22
	F	Yes	No	Vβ2 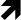 (19%) [D+1]	ND	TSST-1	Vagina (c)	MSSA	3	*tst*	CC30
	F	Yes	No	Vβ2 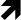 (22%) [D+2]	ND	TSST-1	Vagina (c)	MSSA	3	*tst*; *sea*; *egc*; *selu*^m^	CC30
	F	Yes	Yes (1g/kg)	Vβ2 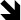 (5.7%) [D+1]	Vβ2 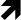 (14.1%) [D+3]	TSST-1	Vagina (c)	MSSA	3	*tst*; *sea*; *egc*; *selu*	CC30
	F	Yes	No	Vβ2 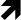 (12%) [D+3]	Vβ2 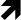 (27.2%) [D+5]	TSST-1	Vagina (c)	MSSA	3	*tst*	CC30
	ccF	Yes	Yes (0.5g/kg)	Vβ2 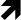 (29.3%) [D+2]	ND	TSST-1	Vagina (c)	MSSA	3	*tst*; sea; egc; *selu*	CC30
Non menstrual staphylococcal toxic shocks	M^n^	Yes	Yes (2g/kg)	Vβ2 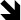 (0.9%) [D+1]	Vβ2 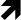 (52.4%) [D+3]	TSST-1	Throat (c)	MSSA	3	*tst*; *sea*; *egc*	CC30
	M	Yes	Yes (2g/kg)	Vβ2 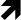 (27.3%) [D+1]	ND	TSST-1	Furuncle (i)^o^	MSSA	3	*tst*; *egc*; *selu*	CC30
	F	Yes	Yes (1g/kg)	Vβ2 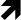 (46.5%) [D+4]	ND	TSST-1	Blood (i)	MSSA	3	*tst*; *sea*; *egc*; *selu*	CC30
	M	Yes	No	Vβ2 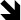 (0.6%) [D+2]	Vβ2 ↔ (9.9%) [D+4]	TSST-1	Nose (c)	MSSA	3	*tst*; *sea*; *egc*; *selu*	CC30
	F	Yes	Yes (1g/kg)	Vβ2 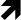 (47.6%) [D+4]	ND	TSST-1	Superficial wound (i)	MSSA	3	*tst*; *sea*; *egc*; *selu*	CC30
	F	Yes	No	ND	ND	ND	Skin (i)	MRSA^p^	2	*tst*; *sec*^q^; *sed*^r^; *selj*^s^*; sell*^t^; *egc*; *selu; ser*^u^	CC5
	F	Yes	No	ND	ND	ND	Skin (i)	MSSA	1	*sec; sell; egc*; *selu*	CC45
	M	No	No	ND	ND	ND	Lung (i)	MSSA	2	*seb; selp*^v^	ND

a *IVIG, Intravenous immunoglobulins*;

b *Vβ alterations of CD3^+^ T cells (%), The expression of 24 main Vβ CD3+ T cells was determined by flow cytometry. Since all our staphylococcal toxic shock syndromes were due to toxic shock syndrome toxin-1 (TSST-1), only Vβ2 repertoire was reported according to it's the only Vβ repertoire targeted by TSST-1. Normal adult range for Vβ2 repertoire according to kit manufacturer, 5.84 to 10.76%*.

c *F, female*.

d *NA, not available*.

e *D, day*.

f *ND, not determined*.

g *TSST-1, toxic shock syndrome toxin−1*.

h *(c): carriage strain*.

i *MSSA, Methicillin Susceptible Staphylococcus aureus* ;

j *tst: gene encoding staphylococcal toxic shock toxin 1*.

k *sea, gene encoding staphylococcal enterotoxin A*.

l *egc, enterotoxin gene cluster encoding staphylococcal enterotoxin G, I, M, N, and O*.

m *selu, gene encoding staphylococcal enterotoxin like U*.

n *M, male*;

o *(i): infection strain*.

p *MRSA, Methicillin Resistant Staphylococcus aureus*.

q *sec, gene encoding staphylococcal enterotoxin C*.

r *sed, gene encoding staphylococcal enterotoxin D*.

s *selj, gene encoding staphylococcal enterotoxin like J*.

t *sell, gene encoding staphylococcal enterotoxin like L*.

u *ser, gene encoding staphylococcal enterotoxin R*.

v **selp, gene encoding staphylococcal enterotoxin like P**.

**Table 5:** VBeta T cell signatures and *Streptococcus pyogenes* toxin gene profiles of patients with streptococcal toxic shock syndrome.

	**Treatments**		**Immunological data**			**Microbiological data**	
**Gender**	**Antitoxin antibiotic (Clindamycin)**	**IVIG^a^ (Dose)**	**Vβ alterations of CD3^+^ T cells (%)^b^**	**Vβ alterations of CD3^+^ T cells (%)**	**Toxin suspected according to Vβ modification profile**	**Site(s) of isolation**	***emm*-types**	**Toxin gene profile**
			**Measurement 1 [day post shock onset]**	**Measurement 2 [day post shock onset]**				
F^c^	Yes	Yes (2 g/kg)	Vβ1 ↔ (4.6%); Vβ2 ↔ (5.8%); Vβ5.1 ↔ (5.6%); Vβ12 ↔ (1.5%); Vβ14 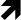 (5.6%) [D^d^+3]	Vβ1 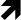 (5.6%); Vβ2 ↔ (9.22%); Vβ5.1 ↔ (4.24%); Vβ12 ↔ (1.32%); Vβ14 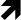 (5.1%) [D^d^+8]	SPEA^e^ or SPEC^f^	Throat	89	*spe*B^g^*, spe*C^h^
M^i^	Yes	No	Vβ1 ↔ (2.2%); Vβ2 ↔ (10.6%); Vβ5.1 ↔ (4.7%); Vβ12 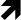 (5.8%); Vβ14 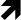 (22.1%) [D+4]	ND^j^	SPEA	Pleural effusion	1	*spe*A^k^ *spe*B
F	Yes	Yes (2 g/kg)	Vβ1 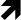 (8.2%); Vβ2 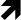 (11.8%); Vβ5.1 ↔ (3.4%); Vβ12 ↔ (1.9%); Vβ14 ↔ (4.4%) [D+3]	ND	SPEC	Blood	87	*spe*B*, spe*C
M	Yes	Yes (2 g/kg)	Vβ1 ↔ (2.3%); Vβ2 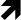 (19.1%); Vβ5.1 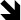 (3.3%); Vβ12 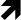 (2.3%); Vβ14 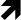 (15.8%) [D+3]	ND	SPEA	Pleural effusion, blood	1	*spe*A, speB
M	Yes	Yes (1 g/kg)	Vβ1 ↔ (2.9%); Vβ2 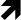 (27.6%); Vβ5.1 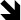 (2.5%); Vβ12 ↔ (1.4%); Vβ14 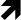 (6.8%) [D+1]	ND	SPEA or SPEC^j^	Pleural effusion, blood	12	*spe*B*, spe*C
F	Yes	Yes (NA^l^)	Vβ1 ↔ (2.5%); Vβ2 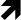 (15.4%); Vβ5.1 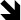 (3.4%); Vβ12 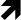 (3.6%); Vβ14 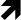 (17.7%) [D+4]	ND	SPEA	Pleural effusion	1	*spe*A, speB
F	Yes	Yes (1 g/kg)	ND	ND	ND	Wound	28	*spe*B*, spe*C
M	Yes	Yes (2 g/kg)	ND	ND	ND	Throat	ND	ND
F	No	No	ND	ND	ND	Blood	ND	ND
F	No	No	ND	ND	ND	Pleural effusion	1	*spe*A*, spe*B*, spe*C
M	Yes	No	ND	ND	ND	Blood	12	*spe*B
F	Yes	No	ND	ND	ND	Pleural effusion, blood	1	*spe*A*, spe*B
M	Yes	No	ND	ND	ND	Tracheal secretions	ND	ND
F	Yes	No	ND	ND	ND	Pleural effusion, tracheal secretions	6	*spe*B*, spe*C
M	Yes	Yes (2 g/kg)	ND	ND^l^	ND	Pleural effusion	1	*spe*A*, spe*B*, spe*C

a *IVIG, Intravenous Immunoglobulin*.

b *Vβ, alterations of CD3^+^ T cells (%): The expression of 24 main Vβ CD3+ T cells was determined by flow cytometry. Only the altered expression of Vβ CD3^+^ T cells is showed according the following normal adult ranges provided by the kit manufacturer: Vβ1: 2.18 to 4.88%Vβ2: 5.84 to 10.76%; Vβ5.1: 3.85 to 7.05%; Vβ12: 1.12 to 2.2%; Vβ14: 2.13 to 4.85%*.

c *F, Female*.

d *D, day*.

e *SPEA, streptococcal pyrogenic exotoxin A*.

f *SPEC, streptococcal pyrogenic exotoxin C*.

g *speB, gene encoding streptococcal pyrogenic exotoxin B*.

h *speC, gene encoding streptococcal pyrogenic exotoxin C*.

i *M, male*.

j *ND, not determined*.

k *speA, gene encoding streptococcal pyrogenic exotoxin A*.

l *NA, not available*.

*No specific other fundings for this study*.

## Discussion

This retrospective analysis of 30 consecutive cases of Staphylococcal and Streptococcal TSS, admitted to the PICUs of the city of Lyon in France, showed differences between groups regarding clinical presentation, origin of infection, and outcome. However, pathophysiological mechanisms involving superantigenic toxins were consistent between groups. We showed that the detection of Vβ profiles could confirm the diagnosis of Staphylococcal and Streptococcal TSS cases, signing a toxin involvement, correlated to the toxin gene profile of the isolated strains.

### Clinical findings

Str-TSS patients were younger than Sta-TSS patients, with 47% of patients younger than 2 years, in agreement with previous studies (2, 4–6). Regarding their clinical signs, TSS associated with para-pneumonic empyema was highly suggestive of a streptococcal origin. Conversely, menstrual Sta-TSS should be suspected in a young adolescent girl with hypotension, rash, gastrointestinal symptoms and fever, whereas non-menstrual Sta-TSS may include a very heterogeneous group of patients in terms of age, gender and comorbidities.

Str-TSS cases had a more severe prognosis than the Sta-TSS cases, including longer PICU stay, higher PIM2 scores, higher number of organ failure with more frequent acute respiratory distress syndrome, and required mechanical ventilation more often and for a longer time, in accordance to UK and Australian studies (5, 6, 40). Although not significant, the case-fatality rate for Str-TSS (20%) was higher than Sta-TSS (7%), that is consistent with the meta-analysis of Chuang et al., who analyzed 27 and 30 studies focused on Sta- and Str-TSS, respectively. They found a 5 to 10% case-fatality rate for Str-TSS, a 3% case-fatality rate for menstrual Sta-TSS and a 5% rate for non-menstrual Sta-TSS (7). Moreover, three later studies that included critically ill children found a case-fatality rate between 20 and 30% for Str-TSS (2, 4, 6); however, that remains below the case-fatality rate (45%) estimated for adults by the French NRC between 2006 and 2010 (1). Recently, Chen et al. reported an absence of death in a large cohort of 62 Staphylococcal and Streptococcal TSS cases (5, 6).

### Factors associated with case-fatality

We found a significantly lower case-fatality rate in patients treated with at least one antitoxin therapy (clindamycin or clindamycin + IVIG) (Table 3). This result was in line with a recent study on TSS-Str demonstrating that clindamycin treatment substantially reduced mortality rate (41, 42). This effect may be enhanced by concurrent treatment with IVIG, contrasting with a previous study suggesting that IVIG had no effect (3, 41). Causality of our results cannot be ascertained because of the retrospective study design with no adjustment for disease severity (i.e., the lack of antitoxin therapy could be due to a misdiagnosis). This supports the need for a large randomized prospective trial, especially to test IVIG. Moreover, this difference in survival rates is likely to be due to the impossibility to receive such urgent treatments in time, because of the very high severity of the disease.

### Microbiological and immunological findings

In accordance with DeVries et al. study which included any positive culture for *S. aureus* (including infection as well as carriage strains) and obtained strain in 72% of the examined cases, we systematically isolated *S. aureus* strains, suggesting a selection bias in our population based on TSS criteria (43). However, in agreement with other studies, bacteremia was rare in Sta-TSS (< 5%) and significantly more frequent in the Str-TSS (40 to 60%) (7). Because characterization of the toxin gene profile of isolates was not mandatory for the inclusion analysis, only 90% were characterized: The 15 isolated *S. aureus* strains belonged to 3 different *agr* groups, and 13/15 carried at least *tst*. Eleven of the *S. aureus* strains were Agr3, and among them, 6 exhibited similar toxin gene profile associating *tst, sea, egc* and *seu*, and belongs to clonal complex 30 (44). These results suggest the presence of a dominant *S. aureus* clone involved in Sta-TSS in France as recently described in United Kingdom. This CC30 MSSA produces more TSST-1 and induces more T-cell proliferation than CC30 MRSA (45). Similarly, Str-TSS were due to a dominant clone of *S. pyogenes* characterized by an *emm*1 gene encoding the M1 protein and *spe*A, in agreement with previous studies (8).

Specific treatments appear most effective when administered early in the development of the disease; thus TSS should be diagnosed as early as possible. However, TSS diagnosis based on the CDC criteria is difficult (43) because some criteria occur lately or rarely, resulting in under-diagnosis of TSS cases (11). Moreover, differential diagnosis with septic shock or Kawasaki syndrome with shock and DRESS syndrome is sometimes challenging because of the association of shock, cutaneous rash and multi organ dysfunctions in these conditions. Parsonnet et al. suggested that the integration of other laboratory criteria such as the isolation of *S. aureus*, the production of TSST 1 (or another superantigen) and the absence of antibodies against superantigen produced by the host could increase the accuracy of TSS diagnosis (46). Based on a biological test measuring functional effects of SAgs on immune system, our study used Vβ profile test to improve TSS diagnosis. As previously described (18), Vβ2 alterations (initial decrease followed by large expansion) correspond to the activation of T cells by TSST-1 and were observed in each of the 12 Sta-TSS cases. Each of the eight Sta-TSS cases, classified as probable according to the CDC criteria and for which Vβ profile was studied, showed a Vβ 2 repertoire expansion similar to other confirmed Sta-TSS cases. Similarly, Vβ expansions corresponded to partial or complete profiles of streptococcal SAgs produced by the clinical isolates were measured in 6/6 patients with Str-TSS. However, in two cases of Str-TSS, the Vβ expansions were difficult to interpret due to partial Vβ profiles when they were compared to the Vβ specificity from the literature (16, 19, 21, 23, 24, 26). These partial profiles may be due to residual neutralizing effects of IVIG infusion before measurement of Vβ repertoire profiles. These discrepancies may also be explained by differences in the methods used to detect Vβ expansions (other sets of antibodies or RT-PCR), the length of the incubation and the use of different cut-off values used to define significant enhancement of Vβ alterations. Ideally, we should determine the Vβ specificity of streptococcal superantigens using a flow cytometric test that has been used for *S. aureus* superantigens (10, 25). Although there is no control group due to the retrospective collection of cases, our study described, for the first time, the use of Vβ profiles to characterize a large pediatric TSS population. This biological test, which was positive in probable as well as confirmed TSS, could identify the toxin involved in the TSS and show alterations at the first measurement, which could help diagnosing or confirming TSS cases. This test may allow for an early (within the first 24–48 h) diagnosis of Sta-TSS, especially in non-menstrual cases, showing a large decrease of Vβ2 associated with severe lymphopenia. Later on (after 48 h), when expansion would confirm the diagnosis whenever the doubt persists; for example when no staphylococcus has been isolated yet.

In contrast, Ferry et al. found no discriminant Vβ signature in septic shock (27), suggesting a high specificity of the Vβ profile for TSS. At least, the Vβ profile might thus constitute a new tool to improve the diagnosis of toxic shock and might also improve the TSS diagnosis criteria of the CDC. However, additional studies are required to evaluate its relevance in partial or doubtful form of TSS cases according to CDC criteria.

### Limitations

The retrospective nature of our study did not allow interpreting reliably the impact of antitoxin therapy on survival. The inability to perform Vβ profile for all patients at a same time point may bias the analysis of the performance of this biological diagnosis test. However, these preliminary results are consistent with those found in adults and require a validation by a prospective multicenter study.

The low sample size did not allow for multifactorial analysis that would have strengthened our findings.

## Conclusion

Sta- and Str-TSS are induced by similar toxins produced by strains with limited diversity which suggests a clonal origin of French strains involved in TSS. However, they differ in their clinical presentations (pulmonary involvement vs. gastrointestinal signs), their source of infection (bacteremia vs. vaginal localization), their severity and their prognosis. The detection of Vβ profiles was helpful for the diagnosis of probable TSS cases and for the identification of toxin involvement but future investigations are required to extensively identify the Vβ profiles of streptococcal toxins. However, this test might be added to the TSS diagnosis criteria in the future. Antitoxin therapies (clindamycin and/or IVIG) were associated with a better survival. However, a potential bias related to the discrepancy in the timing of treatment delivery may limit the interpretation of these results. The conduct of a large randomized controlled trial is mandatory to assess IVIG and anti-toxin chemotherapy efficacy.

## Author contributions

EJ participated in study design, data analysis and interpretation, critically revised and approved the final manuscript as submitted. P-AB participated in the study design, data analysis, and interpretation and wrote the article as submitted. CJ participated in the study design, acquisition of data, statistical analysis, and interpretation, wrote the article and approved the final manuscript as submitted. GL participated in the study design, critically revised, and approved the final manuscript as submitted. CB performed some immunological analyses and approved the final manuscript as submitted. CP helps to analyze data from streptococcal strains and approved the final manuscript as submitted. AP participated in the study design, acquisition of data, statistical analysis and interpretation, and approved the final manuscript as submitted. AT, FL, and MB analyzes data from staphylococcal strains and approved the final manuscript as submitted. FV and YG revised and approved the final manuscript as submitted. OD participated in the study design, patient recruitment, data analysis and interpretation, wrote the article, and approved the final version of the manuscript as submitted. All authors approved the final manuscript as submitted and agree to be accountable for all aspects of the work.

### Conflict of interest statement

The authors declare that the research was conducted in the absence of any commercial or financial relationships that could be construed as a potential conflict of interest.
